# A Rapid Colorimetric Method for Determining Calcium, Inorganic Phosphorus, and Zinc in Human Milk Using Clinical Laboratory Reagents

**DOI:** 10.1002/jcla.70146

**Published:** 2025-12-21

**Authors:** Miori Tanaka, Misaki Mochida, Midori Date, Katsumi Mizuno

**Affiliations:** ^1^ The Nippon Foundation Human Milk Bank Tokyo Japan; ^2^ Department of Pediatrics Showa Medical University School of Medicine Tokyo Japan

**Keywords:** calcium, colorimetric assay, human milk, inorganic phosphorus, zinc

## Abstract

**Background:**

Calcium (Ca), inorganic phosphorus (IP), and zinc (Zn) are essential minerals in human milk for infant growth and health. Current methods for determining minerals in human milk, including inductively coupled plasma atomic emission spectroscopy (ICP‐AES), are time‐consuming and laborious. This study aimed to measure Ca, IP, and Zn in human milk by a rapid, simple, and accurate method using clinical laboratory reagents based on a colorimetric assay.

**Methods:**

71 pooled donor human milk (DHM) samples were collected after Holder pasteurization. The only pretreatment required was dilution, and samples were diluted 5‐fold. Ca, IP, and Zn concentrations were measured by colorimetric assay using an automated analyzer. The results were validated in terms of repeatability, recovery, and comparison with ICP‐AES.

**Results:**

The coefficients of variation for intra‐assay precision were 0.7%–1.9% for Ca, 1.5%–2.1% for IP, and 0.8%–7.4% for Zn. The recovery rates ranged from 104.1% to 104.9% for Ca, 96.3%–103.4% for IP, and 94.5%–103.2% for Zn with high, medium, and low concentrations. There were significant correlations between Ca, IP, and Zn levels determined by colorimetric assay and Ca, total P, and Zn levels determined by ICP‐AES (Ca: *r* = 0.944, IP: *r* = 0.463, and Zn: *r* = 0.949, *p* < 0.001, *n* = 58).

**Conclusion:**

These results confirm the precision and accuracy of the colorimetric assay using clinical laboratory reagents for determining Ca, IP, and Zn levels in human milk. Its use may contribute to the rapid and easy provision of appropriate DHM to preterm infants.

## Graphical abstract

We evaluated a colorimetric assay using clinical reagents for determining Ca, IP, and Zn in human milk. Significant correlations were observed between Ca, IP, and Zn levels determined by colorimetric assay and Ca, total P, and Zn levels determined by ICP‐AES. This rapid and simple method may support clinical monitoring of donor milk composition.

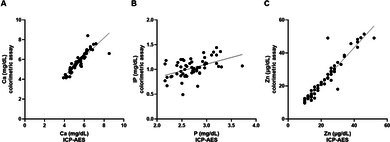

## 1 Introduction

Human milk contains more than 20 minerals, including calcium (Ca), phosphorus (P), and zinc (Zn). Ca and P are involved in various biological processes, such as bone mineralization and cell signaling [1]. Ca concentration decreases linearly over the duration of lactation, with a mean Ca concentration in human milk of 261 mg/L based on 154 studies [2]. The concentrations of total P and inorganic phosphorus (IP) in human milk are 120–150 mg/L [3] and 40–70 mg/L [4, 5], respectively. Rickets, a common metabolic bone disease in infants and young children, is usually secondary to deficiencies of Ca or P [6]. Zn plays a crucial role in enzyme function, gene transcription, and cell signaling [7]. Zn levels in milk are initially high and then decrease rapidly to reach a plateau, and a systematic review and meta‐analysis of 242 studies found a mean Zn concentration in human milk of 2.57 mg/L [2]. Zn deficiency in infants results in growth disorders and impaired immune function, leading to increased morbidity and mortality from diarrhea and respiratory infections [8]. Because of these fundamental biological roles, Ca, P, and Zn are recognized as important nutrients for infants.

Preterm infants are especially at increased risk of mineral deficiencies due to higher nutritional requirements and limited endogenous stores [9]. Most fetal accretion of Ca, P, and Zn occurs in the third trimester of pregnancy (after 28 weeks of gestation), and preterm infants may thus partially or completely avoid the optimal stage for acquiring these nutrients [10, 11]. Donor human milk (DHM) from human milk banks is widely recommended for preterm infants when the mother's own milk is not available [12]. Notably, the use of DHM instead of formula has been shown to reduce the occurrence of necrotizing enterocolitis in preterm infants by about half [13]. The composition of human milk is influenced by various factors, and negative relationships between postpartum week and Ca, P, and Zn levels in human milk have been investigated [2, 14]. Nutrient variability in DHM may contribute to growth deficits in preterm infants, and DHM may need to be fortified with additional nutrients, such as protein and minerals, in order to meet the high requirements of preterm infants [15]. Japanese human milk banks thus measure the nutrient composition (macronutrients, minerals, and immune components) of all DHM.

Various analytical methods have been used to measure minerals in human milk, including fluorescence, atomic absorption spectroscopy (AAS), inductively coupled plasma atomic emission spectroscopy (ICP‐AES), and inductively coupled plasma mass spectrometry (ICP‐MS) [16]. The most commonly used method to determine Ca and Zn in human milk is AAS, followed by ICP‐MS and ICP‐AES [2], while the spectrophotometric molybdenum blue method has also been used to determine P levels in dairy products [17]. In Japan, the Consumer Affairs Agency specifies ICP‐AES as the prescribed method for analyzing Ca, P, and Zn in food products, and previous studies have thus utilized ICP‐AES for measuring these elements in human milk [18, 19]. The above methods, however, require special instrumentation and large amounts of milk, involve the use of harmful and corrosive chemicals, and are time‐consuming and laborious.

In clinical practice, Ca, IP, and Zn levels in serum are measured as part of routine blood analyses; however, there are no clinical laboratory reagents available to assay minerals in human milk. Although a previous report described a semi‐automated method for analyzing Ca and P in small volumes of human milk using commercially available kits for serum samples [20], this approach still requires complicated pretreatment steps, including ashing followed by digestion with dilute hydrochloric acid to ensure that the milk is translucent before colorimetric analysis. The current study aimed to validate the use of clinical laboratory reagents, typically used for serum and urine analyses, for the measurement of Ca, IP, and Zn in human milk, in terms of repeatability, recovery, and comparison with ICP‐AES. These reagents are used for colorimetric analysis, based on the activation of enzymes or chelate reaction, with sample dilution as the only required pretreatment step.

## 2 Materials and Methods

### 2.1 Milk Samples

This study was conducted at The Nippon Foundation Human Milk Bank (TNFHMB) and approved by the Showa Medical University Research Ethics Review Board (approval number: 2714). Human milk samples were collected using a convenience sampling approach from 71 donors registered with TNFHMB who met the eligibility criteria. Milk samples were included regardless of maternal age, the age of the child, or other donor characteristics. The donor registration requirements included the ability to produce more human milk than what her child needs. Exclusion criteria included a history of blood transfusions or organ transplants and a history of treatment for malignancies such as leukemia or lymphoma in the past 3 years. All donors had completed a screening process consisting of a health questionnaire and a medical interview with doctors or midwives who reviewed the health questions and assessed the donor's suitability. Blood screening tests for the human immunodeficiency virus (HIV I/II), the human T‐cell leukemia virus (HTLV I/II), hepatitis B and C, and syphilis were then undertaken. Milk was obtained by hand or pump expression, according to each donor's usual practice. After expression, milk in clean bags was frozen and stored in a freezer at the donor's home. The milk was then transported from the donor's home to TNFHMB or the Japan Human Milk Bank Association (JHMBA) via refrigerated transport, to ensure a cold transport chain. On arrival at TNFHMB or JHMBA, milk was stored at −30°C until pasteurization. Frozen milk was thawed in a refrigerator overnight and then pasteurized within 24 h. In preparation for pasteurization, the thawed milk was pooled with other milk from the same donor to reduce nutrient variability. Holder pasteurization (62.5°C, 30 min) was performed with a Sterifeed S90 pasteurizer (MediCare Colgate, Kentisbeare, UK), Barkey clinitherm pasteur 10/80 (Barkey, Leopoldshöhe, Germany), or Racoon dry pasteurizer HMP‐4 (Mita Rika Kogyo, Osaka, Japan). Milk samples were collected after pasteurization and stored at −80°C until the respective tests. All donors provided written consent for the use of their human milk for research purposes.

### 2.2 Reagents

Accuras Auto Ca II (Reagents 1 and 2), Accuras Auto IP (Reagents 1 and 2), Accuras Auto Zn (Reagents 1 and 2), calibrators of Ca, IP, and Zn, Aalto Control LEVEL I α, Aalto Control LEVEL II α, Zn control, and diluted solution for human milk were obtained from Shino‐Test (Tokyo, Japan).

### 2.3 Ca Analysis Procedure

Ca levels were determined using the Accuras Auto Ca II based on increased phospholipase D (PLD)‐catalyzed hydrolysis of bis (*p*‐nitrophenyl) phosphate (BPNPP) by Ca^2+^ [21]. The assay is a 2‐point fixed‐rate assay performed at 37°C. Milk samples were diluted 5‐fold with a diluted solution. Measurements were performed automatically using a CA‐270 Clinical Chemistry Analyzer (Furuno Electric, Hyogo, Japan) as follows: 6.6 μL of diluted milk sample and 150 μL of PLD solution (Reagent 1) were pipetted into a cuvette and, after 5.3 min, 75 μL of BPNPP solution (Reagent 2) was added. The absorbance of *p*‐nitrophenol released by the reaction was measured at 415 nm 2.1 min and 5 min after adding Reagent 2, and the rate of increase in absorbance was calculated. The concentration of Ca in the milk samples was quantified by comparison with a spline calibration curve obtained from Ca calibrator. We checked that the concentrations of Aalto Control LEVEL I α and Aalto Control LEVEL II α were within range before measuring human milk samples.

### 2.4 IP Analysis Procedure

IP levels were determined using the Accuras Auto IP based on the reaction of purine nucleoside phosphorylase (PNP) coupled with xanthine dehydrogenase (XDH) [22]. The assay is a 2‐point end assay performed at 37°C. Milk samples were diluted 5‐fold with a diluted solution. Measurements were performed automatically using a CA‐270 Clinical Chemistry Analyzer as follows: 2.5 μL of diluted milk sample and 150 μL of PNP and XDH solution (Reagent 1) were pipetted into a cuvette and after 5 min, the absorbance was measured at 340 nm. After 19 s, 50 μL of inosine and oxidized form of nicotinamide adenine dinucleotide (NAD^+^) solution (Reagent 2) was added. The released nicotinamide adenine dinucleotide hydride (NADH) was then measured at 340 nm 5 min after adding Reagent 2, and the changes in absorbance were calculated. The concentration of IP in the milk samples was quantified by comparison with a spline calibration curve obtained from IP calibrator. We checked that the concentrations of Aalto Control LEVEL I α and Aalto Control LEVEL II α were within range before measuring human milk samples.

### 2.5 Zn Analysis Procedure

Zn levels were determined using the Accuras Auto Zn based on the chelation of Zn^2+^ with 2‐(5‐Bromo‐2‐pyridylazo)‐5‐[*N*‐propyl‐*N*‐(3‐sulfopropyl)amino]phenol (5‐Br‐PAPS) [23]. The assay is a 2‐point end assay performed at 37°C. Milk samples were diluted 5‐fold with a diluted solution. Measurements were performed automatically using a CA‐270 Clinical Chemistry Analyzer as follows: 10 μL of diluted milk sample and 150 μL of buffer solution (Reagent 1) were pipetted into a cuvette and, after 5 min, the absorbance was measured at 546 nm. After 19 s, 50 μL of 5‐Br‐PAPS solution (Reagent 2) was added. The absorbance of the chelate compound released by the reaction was then measured at 546 nm 5 min after adding Reagent 2, and the changes in absorbance were calculated. The concentration of Zn in the milk samples was quantified by comparison with a spline calibration curve obtained from the Zn calibrator. We checked that the concentrations of Zn control were within range before measuring human milk samples.

### 2.6 Precision

To evaluate precision, a repeatability (intra‐assay) study was carried out within a day. 20 replicate measurements were performed using 9 human milk samples with high, medium, and low concentrations of Ca, IP, or Zn.

### 2.7 Recovery Test

We supplemented 9 human milk samples with reference standards for Ca, IP, and Zn, respectively (Shino‐Test), and measured the Ca, IP, or Zn levels. The percentage of recovery was calculated as follows:

$$\text{Recovery}=\left(\text{C1}-\text{C2}\right)/\left(\text{C3}-\text{C4}\right)\times 100\%$$

C1: Sample concentration after adding reference standard.

C2: Sample concentration before adding reference standard.

C3: Concentration of adding reference standard.

C4: Concentration of a diluted solution.

### 2.8 Comparison With ICP‐AES

Ca, IP, and Zn levels in 58 human milk samples determined by colorimetric assay were compared with Ca, total P, and Zn levels measured by ICP‐AES. The ICP‐AES analyses were outsourced to the Japan Food Research Laboratories (Tokyo, Japan).

### 2.9 Statistical Analysis

Statistical analyses were performed using the GraphPad Prism 10 software (GraphPad Software, La Jolla, CA, USA). All continuous variables were tested for normality by a D'Agostino–Pearson test. The relationship between the colorimetric assay and ICP‐AES results was determined using Spearman's rank correlation coefficient because the data exhibited a nonparametric distribution. Simple linear regression analysis was performed to examine the relationship between 2 variables. Differences were considered statistically significant when *p* < 0.05.

## 3 Results

### 3.1 Precision

We estimated the intra‐assay precision from 20 replicate analyses for Ca, IP, and Zn, respectively, in 9 human milk samples. The mean, standard deviation (SD), and coefficient of variation (CV) for each sample are shown in Table 1. The CVs ranged from 0.7% to 1.9% for Ca, 1.5%–2.1% for IP, and 0.8%–7.4% for Zn, which are considered to indicate good precision for a routine method.

**TABLE 1:** Intra‐assay precision of colorimetric assay (*n* = 20).

Sample		Mean	SD	CV (%)
Ca	High	10.2	0.07	0.7
	Medium	6.3	0.05	0.8
	Low	3.9	0.08	1.9
IP	High	1.4	0.02	1.5
	Medium	1.1	0.02	1.5
	Low	0.7	0.01	2.1
Zn	High	53.9	0.41	0.7
	Medium	13.9	0.36	2.6
	Low	5.7	0.42	7.4

Abbreviations: Ca = calcium; CV = coefficient of variation; IP = inorganic phosphorus; SD = standard deviation; Zn = zinc.

### 3.2 Recovery Test

We calculated the percentage of recovery by measuring Ca, IP, and Zn concentrations, respectively, in 9 human milk samples before and after adding the reference standard. As shown in Table 2, the recovery rates were between 104.1% and 104.9% for Ca, 96.3% and 103.4% for IP, and 94.5% and 103.2% for Zn.

**TABLE 2:** Recovery test for colorimetric assay.

Sample		C1	C2	C3	C4	Recovery (%)
Ca	High	9.6	7.3	3.1	0.9	104.1
	Medium	7.8	5.6	3.0	0.9	104.7
	Low	6.3	3.9	3.2	1.0	104.9
IP	High	2.6	1.2	1.3	0	101.4
	Medium	2.2	0.8	1.3	0	103.4
	Low	1.9	0.5	1.4	0	96.3
Zn	High	70.4	53.6	17.2	1.0	103.2
	Medium	27.8	12.8	16.3	1.3	99.7
	Low	20.9	7.2	15.9	1.5	94.5

*Note:* Recovery = (C1 − C2)/(C3 − C4) × 100%. C1: Sample concentration after adding reference standard. C2: Sample concentration before adding reference standard. C3: Concentration of adding reference standard. C4: Concentration of a diluted solution.

Abbreviations: Ca = calcium; IP = inorganic phosphorus; Zn = zinc.

### 3.3 Comparison With ICP‐AES

**FIGURE 1:**
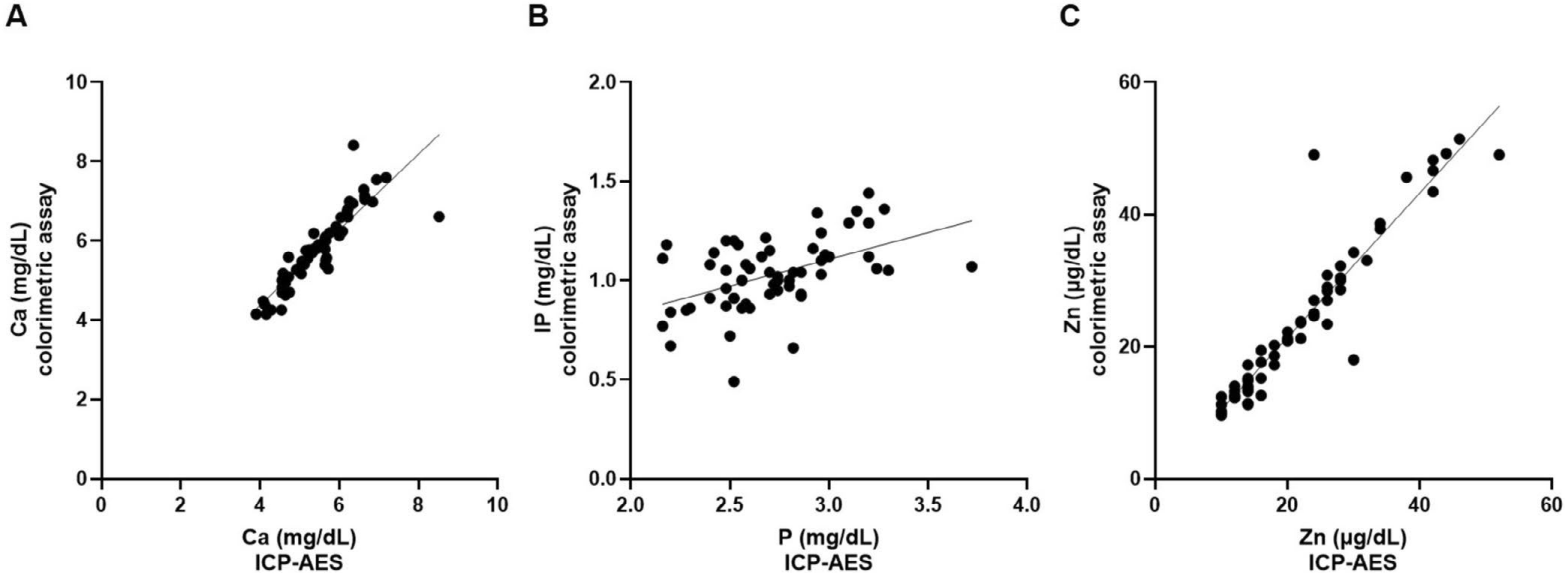
Correlation between colorimetric assay and ICP‐AES for Ca, IP, and Zn determination in human milk. (A) Ca levels in 58 human milk samples were measured by colorimetric assay and ICP‐AES (*y* = 0.952*x* + 0.568, *r* = 0.944, *p* < 0.001). (B) IP and total P levels in 58 human milk samples were measured by colorimetric assay and ICP‐AES (*y* = 0.269*x* + 0.299, *r* = 0.463, *p* < 0.001). (C) Zn levels in 58 human milk samples were measured by colorimetric assay and ICP‐AES (*y* = 1.093*x* − 0.488, *r* = 0.949, *p* < 0.001).

We analyzed 58 human milk samples by colorimetric assay (*y*) and ICP‐AES (*x*). As shown in Figure 1, a strong correlation was observed between Ca and Zn levels measured by colorimetric assay and those measured by ICP‐AES (Ca: *r* = 0.944, *p* < 0.001; Zn: *r* = 0.949, *p* < 0.001). The regression equations were *y* = 0.952*x* + 0.568 for Ca and *y* = 1.093*x* − 0.488 for Zn. IP levels determined by colorimetric assay were positively correlated with total P levels determined by ICP‐AES, but the correlation coefficient for IP was lower than those for Ca and Zn (*r* = 0.463, *p* < 0.001), with a regression equation of *y* = 0.269*x* + 0.299.

## 4 Discussion

Ca, IP, and Zn are essential nutrients for growth and health during infancy and early childhood, and human milk is an important source of these elements. Current methods for determining mineral levels in human milk are time‐consuming and laborious, making them difficult to use for routine analysis. Colorimetric assay measures the intensity of colored compounds generated by chemical reactions using a spectrophotometer. Although this method is relatively inexpensive and generally does not require extensive specialized training, it may necessitate meticulous sample pretreatment, including ashing and subsequent digestion with dilute hydrochloric acid, to eliminate the effects of the organic matrix in human milk. Human milk has a more complex matrix than serum and urine, containing not only protein but also fat and carbohydrate. Like iron and copper, Ca and Zn can be found in both the whey and fat fractions of human milk [24]. Human milk also contains several metal‐binding proteins, such as albumin and casein, which bind to Ca^2+^, Zn^2+^, and other metal cations [25, 26]. The present study validated the application of clinical laboratory reagents used for serum and urine analyses to develop a rapid, accurate, and simple method for the detection of Ca, IP, and Zn in human milk. This colorimetric method simplifies mineral determination in human milk, requiring only sample dilution and no complex pretreatment with surfactants in the diluted solution to make the milk translucent.

The validation study demonstrated good precision for a routine method, with CVs for the repeatability assay of 0.7%–1.9% for Ca, 1.5%–2.1% for IP, and 0.8%–7.4% for Zn in human milk with high, medium, and low concentrations. To determine the inhibitory effect of the matrices in human milk on the enzyme and chelate reactions, we also performed a recovery test. The respective recovery rates for Ca, IP, and Zn in human milk were 104.1%–104.9%, 96.3%–103.4%, and 94.5%–103.2%, with high, medium, and low concentrations, suggesting that the matrix effect on chemical reactions in human milk was minimal. For Ca measurement, PLD is specifically activated by Ca, not by magnesium, which activates other Ca‐detecting enzymes [27]. Furthermore, the affinity of Ca for PLD is much greater than its affinity for albumin [21], allowing this reagent to react with albumin‐bound Ca. For Zn measurement, the surfactant in Reagent 1 liberates Zn from proteins, enabling the complete quantification of protein‐bound Zn. Finally, correlation analysis between ICP‐AES and colorimetric assay showed a significant correlation for all three minerals analyzed. The correlations for Ca and Zn were particularly strong, exhibiting high correlation coefficients and regression slopes close to 1. These results confirm the precision and accuracy of this colorimetric assay using clinical laboratory reagents for measuring Ca, IP, and Zn in human milk.

P in human milk is a mixture of organic and inorganic forms, with various organic P such as casein‐bound P and phospholipids [28, 29]. Although blood tests usually measure IP, many studies on human milk have focused on total P, and there is thus no established method for quantifying IP in human milk. PNP, the enzyme used in our reagent, does not react with organic P, such as adenosine triphosphate [30]. The high recovery rates for IP in this study and the average IP/total P percentage (37.7%), which aligned closely with previous results (35.9%) [31], indicated that the current colorimetric method represents an accurate tool for measuring IP concentrations in human milk. Although the correlation between IP measured by colorimetric assay and total P measured by ICP‐AES was significant in this study, the correlation coefficient was lower than those for Ca and Zn. This might be attributed to the variability in the ratio of total P and IP in human milk depending on lactation stage and individual differences [31]. The rate of absorption of IP is over 90%, whereas that of organic P is about 40%–60% [32]. Additionally, most P added to human milk fortifiers is IP, including calcium glycerophosphate and inorganic salts [33]. Concomitant with our results showing that higher IP levels corresponded to higher total P levels in human milk, we believe that determining IP levels in human milk and DHM by this simple colorimetric method represents a clinically significant finding. To clarify the importance of IP in the nutrition of preterm infants, it will be necessary to examine the relationship between IP intake and infant growth and long‐term outcomes.

Ca, P, and Zn concentrations decrease sharply from colostrum to transitional milk, followed by a gradual decline throughout lactation [1, 2, 14]. Human milk banks provide several types of DHM obtained from donors at different postpartum weeks, with varying mineral contents; however, mineral variability in DHM may represent a major challenge in ensuring adequate postnatal growth. Nutritional analysis of DHM would support the allocation of the most appropriate DHM to each preterm infant, such as by assigning DHM with adequate mineral concentrations to infants requiring long‐term DHM feeding. Due to the low mineral content of human milk, it alone is insufficient to meet the estimated needs of preterm infants [34]. In neonatal intensive care units, human milk fortifiers are added to DHM to supplement nutrients in short supply, particularly protein, Ca, and P [15]. Because individualized fortification has been demonstrated to improve nutrient intake, physical and psychosocial development in preterm infants compared with standard fortification [15, 35], individualized fortification based on nutritional analysis may help to optimize the mineral contents of DHM and thus improve infant growth. Colorimetric mineral analysis using clinical laboratory reagents and an automated analyzer is rapid, involves no complicated steps, reduces human error, and requires minimal sample volumes. Sample dilution is the only required pretreatment step before adding the reagents to the analyzer, thus eliminating the use of hazardous chemicals and complex pretreatment processes required for conventional methods. Furthermore, these reagents can be easily adapted for use in automated analyzers commonly available in hospitals, leading to rapid clinical decisions on DHM selection and neonatal care. Therefore, this rapid and simple method is considered suitable for routine measurements of Ca, IP, and Zn in human milk.

A limitation of this study is that only pasteurized and frozen DHM samples were analyzed; however, the concentrations of minerals including Ca, P, and Zn were reported to be largely preserved after freezing and pasteurization [36, 37, 38]. In addition, linearity could not be experimentally verified in human milk samples because 5‐fold dilution is required to make the milk translucent before analysis. Most human milk samples contain approximately 20–30 mg/dL of Ca, which becomes 4–6 mg/dL after 5‐fold dilution; further serial dilution would reduce the concentration below the detection limit (0.2 mg/dL). Similar limitations apply to IP and Zn. The manufacturer has demonstrated good linearity for Ca, IP, and Zn using reference standards within the detection range.

In conclusion, the reported colorimetric assay is a rapid and simple method for determining Ca, IP, and Zn levels in human milk and correlates well with the conventional ICP‐AES method. This assay is expected to enable the timely and convenient provision of the appropriate DHM for each preterm infant and to facilitate individualized mineral supplementation.

## Author Contributions

M.T. designed the study; M.M. and M.D. collected the data; M.T. and M.M. analyzed the data; M.T. and M.M. wrote the manuscript; K.M. supervised the study. All authors read and approved the final manuscript.

## Funding

The authors have nothing to report.

## Ethics Statement

Ethical approval was obtained from Showa Medical University Research Ethics Review Board (Approval number: 2714, 21 June 2021). All donors provided written consent for the use of their human milk for research purposes.

## Conflicts of Interest

The authors declare no conflicts of interest.

## Data Availability

The datasets used and/or analyzed during the current study are available from the corresponding author on reasonable request.
